# Anti-Inflammatory, Anti-Arthritic and Anti-Nociceptive Activities of *Nigella sativa* Oil in a Rat Model of Arthritis

**DOI:** 10.3390/antiox8090342

**Published:** 2019-08-25

**Authors:** Cinzia Nasuti, Donatella Fedeli, Laura Bordoni, Marco Piangerelli, Maurizio Servili, Roberto Selvaggini, Rosita Gabbianelli

**Affiliations:** 1 School of Pharmacy, Pharmacology Unit, University of Camerino, 62032 Camerino, MC, Italy; 2 School of Pharmacy, Molecular Biology Unit, University of Camerino, 62032 Camerino, MC, Italy; 3 School of Science and Technology, Computer Science Division, University of Camerino, 62032 Camerino, MC, Italy; 4 Department of Agricultural, Food and Environmental Sciences, University of Perugia, 06121 Perugia, Italy

**Keywords:** *Nigella sativa*, adjuvant arthritis, inflammation, IL-6, clustering

## Abstract

This study investigated the preventive efficacy of the crude oil extracted from *Nigella sativa* seeds in a rat model of arthritis induced by using complete Freund’s adjuvant (CFA). *Nigella sativa* oil at 1.82 mL/kg or 0.91 mL/kg (corresponding to 1596 and 798 mg/kg, respectively) was orally administered for 25 days from the day of immunization. One immunized group was treated orally with indomethacin (3 mg/kg) as a reference drug. Body weight growth rate, paw swelling, arthritis score, mechanical allodynia, locomotor activity and anxiety-like behavior were observed, and the levels of Interleukin 6 (IL-6), C-reactive protein, albumin and total cholesterol in plasma were measured on days 15 and 25. *Nigella sativa* oil showed anti-inflammatory, anti-arthritic and anti-nociceptive activities that were significant as compared to untreated arthritic rats but less than indomethacin. These results indicated that *Nigella sativa* oil significantly attenuated adjuvant-arthritis in rats and the higher dose (1.82 mL/kg) prevented the development of arthritis with an inhibition of 56%.

## 1. Introduction

Rheumatoid arthritis (RA) is a chronic, inflammatory, systemic autoimmune disease that is characterized by synovial membrane inflammation, swelling, autoantibody production and cartilage and bone destruction affecting approximately 1% of the world population [1]. RA more commonly affects the aged population, and is associated with systemic complications, disability, early mortality and socioeconomic costs [2].

Research on RA employs rodent models where immune-mediated arthritis can be induced by mycobacterium (i.e., adjuvant-induced arthritis, AIA) or collagen (i.e., collagen-induced arthritis, CIA) [3]. The main difference between them is that AIA is induced by substances that do not contain a defined self-antigen [4]. AIA is a disease produced in rats by immunization with a complete Freund adjuvant (CFA) composed of killed *Mycobacterium tuberculosis* and characterized by a rapid onset and progression to polyarthritis. Primary arthritic lesions appear immediately from day 0 in the injected hind paw followed by the development of secondary arthritic lesions in the controlateral hind paw at around day 15 from the injection of CFA.

AIA is a neutrophil and T cell-dependent disease leading to joint swelling and loss of its function, bone resorption and cartilage degradation with similar features observed in human RA [5]. Pro-inflammatory cytokines expressed in the joint during the early stages of inflammation include interleukin-17 (IL-17), interferon-γ (IFN-γ), tumor necrosis factor-α (TNF-α) and interleukin-1β (IL-1β). As inflammation progresses in the joint, increased levels of interleukin-6 (IL-6) can be detected in the plasma showing to be involved especially in the later phase of AIA [6].

Common drugs administered for pain relief and delay of RA progression include nonsteroidal anti-inflammatory drugs (i.e., indomethacin and acetaminophen), disease-modifying antirheumatic drugs (i.e., methotrexate and leflunomide), hormone drugs (i.e., corticosteroids) and biological agents (i.e., abatacept and rituximab) [7]. Furthermore, combination of these drugs is the major strategy for the treatment of RA. However, these drugs are expensive and result in side effects after long-term use, such that nonsteroidal anti-inflammatory drugs induce gastric injury or corticosteroids induce infections and bone fractures, thus limiting their application. In this regard, anti-inflammatory substances from various sources like medicinal plants used in traditional medicine have aroused great interest in recent years due to their therapeutic potential. One of them is thymoquinone, which is the main bioactive component of the black seeds of *Nigella sativa*, a plant native to Asian and Arabic countries belonging to the Ranunculaceae family. Many pharmacological actions such as antioxidant, antimicrobial, anti-inflammatory, immunomodulator, analgesic, anti-arthritic, anti-diabetic, anti-asthmatic and anti-cancer have been attributed to thymoquinone and, at least in part, to the other compounds present in *Nigella sativa* [8,9]. In a previous in vitro study, we demonstrated the antioxidant and anti-inflammatory properties of a crude oil extracted from the seeds of *Nigella sativa* produced in the Marche region (Italy) [10].

The purpose of the present study was to evaluate the analgesic and anti-inflammatory properties of *Nigella sativa* oil produced in the Marche region (Italy) [10], in a rat AIA model over a 25-day period. Specifically, we used rats with CFA-induced arthritis to mimic human RA, as it produces systemic inflammation that results in severe joint swelling and remodeling. Our specific aims were to (1) evaluate the safety of *Nigella sativa* oil by acute toxicity study, (2) determine the anti-arthritic and anti-inflammatory effects of *Nigella sativa* oil by measuring paw edema, % inhibition of paw edema and arthritis score, (3) evaluate the anti-nociceptive effects of *Nigella sativa* oil and (4) assess the effect of *Nigella sativa* on plasma biomarkers by examining levels of IL-6, C-reactive protein (CRP), albumin and total cholesterol (TC).

## 2. Materials and Methods 

### 2.1. Materials

CFA at a concentration of 10 mg/mL was purchased from Chondrex (Biozol, Munchen, Germany). IL-6 and rat-CRP ELISA kits were purchased from Invitrogen (Monza, Italy). TC and albumin assay kits were generously donated by Chema Diagnostica (Monsano, Italy). Indomethacin powder was provided by Alfasigma (Pomezia, Italy). All other chemicals and reagents were commercially available and of analytical grade.

### 2.2. Nigella sativa Oil Preparation

*Nigella sativa* oil was extracted from the seeds of *Nigella sativa L*. cultivar, also known as black cumin, produced in the Marche region of Italy by the Tre Ponti Snr factory (Polverigi, Italy). Cold pressing of *Nigella sativa* seeds by means of a squeezing machine (Vero Energia Italia S.r.l., Ravenna, Italy) was employed to produce the oil. The oil was filtered to remove solid residues 10 days after extraction, and it was stored in a box protected from heat and sunlight before use.

The concentration of thymoquinone, the predominant constituent of *Nigella sativa* fixed oil was 7.2 mg/mL, as previously reported [10].

### 2.3. Quantitative Analysis of Fatty Acids and Tocopherols in Nigella sativa Oil 

Fatty acid composition of *Nigella sativa* oil samples was determined by gas chromatography (GC) using a GC Dani Master GC equipped with a flame ionization detector (FID; Cologno Monzese, MI, Italy), according to the official method of the European Commission [11]. The following operating conditions were set: A Select Fatty Acid Methyl Esters (FAME) capillary column 100 m × 0.25 mm (Agilent Technologies, Santa Clara, CA, USA) was employed and the initial oven temperature was set to 140 °C (maintained for 5 min), then raised to 240 °C with an increase of 4 °C/min (maintained for 10 min); the total running time was 40 min. Helium was used as a gas carrier at a constant flow of 1.0 mL/min. Both detector and injector temperatures were 260 °C. The injection volume was 1 μL with the injector set in split mode (1:50 split ratio). The resulting fatty acid content is shown as a percentage of the peak area compared to the total area using a standard mixture of fatty acids for their identification (retention time of fatty acids in the oil was compared with those of a known standard mixture (Supelco 37 Component FAME Mix, Sigma-Aldrich, Milan, Italy).

Tocopherols were evaluated by high performance liquid chromatography (HPLC) analysis with an HPLC system (Agilent Technologies Model 1100) using an excitation wavelength of 294 nm and an emission wavelength of 300 nm. The HPLC system was equipped with a diode array detector (DAD), and a fluorescence detector (FLD), according to the procedure reported in Esposto et al. [12]. Data are expressed as mean ± standard deviation of two replicates.

### 2.4. Animals

Sixty-five female Wistar rats (60 days old) weighing 150–180 were purchased from Charles River (Calco, LC, Italy). Paired rats were lodged in a room at constant temperature (21 ± 5 °C), humidity (45–55%) and artificial 12:12 h light/dark cycle (lights off at 8:00 a.m.). Rats were fed food and water ad libitum in their home cages. Following one week of animal acclimatization in these laboratory conditions, the experiments were performed during the nocturnal phase of the light/dark cycle.

European Guidelines (Directive 2010/63/EU) for the Care and Use of Laboratory Animals were strictly followed and all efforts were made to minimize suffering of animals. The procedure was permitted by the Italian Ministry of Health (Protocol Number: 393/2016-PR).

### 2.5. Acute Toxicity Study

The oral acute toxicity study of *Nigella sativa* oil was evaluated according to the Organization for Economic Co-operation and Development (OECD) guideline 423 [13] on six female nulliparous Wistar rats (180–200 g). Since previous evidences on *Nigella sativa* oil suggested that mortality was unlikely at the highest starting dose level, the plant extract was administered orally with an initial dose of 2000 mg/kg body weight corresponding to 2.28 mL/kg body weight. The mortality was measured after 24 h, and a toxic dose was considered that which lead to the death of 2/3 or 3/3 of rats. Experiments were repeated with the same dose when only one of three rats died. To calculate the dose of oil to be administered, the body weight of each rat was measured before each experiment.

Survival was monitored continuously during the first 24 h and once a day over the next 13 days. Daily, animals were observed for any toxic effect on body weight, behavior, respiration, convulsion, tremor, temperature, salivation, diarrhea, lethargy and eye and skin colors. At the end of the experiment (14 days), all surviving animals were euthanized by carbon dioxide inhalation and subjected to gross necropsy, which included careful examination of liver, kidneys, lung, thymus, spleen, brain, heart, ovaries, intestines and adrenals. The value of the oral lethal dose 50 (LD50) was identified as reported by the OECD guideline 423.

### 2.6. CFA-Induced Arthritis Model

Forty-eight animals were anesthetized with 3% isoflurane in oxygen and arthritis was induced by a single intradermal injection of CFA (0.01 mL) into their right hind metatarsal foot-pad. Rats of the control group (*n* = 11) were anesthetized and injected with an equal volume of saline instead of CFA, then treated orally with saline (1.82 mL/kg), once a day, for 25 days. CFA-treated rats (*n* = 12 per group) were divided into four groups and gavaged daily for 25 days: The first group (CFA + *Nigella sativa* 0.91 mL/kg) received 0.91 mL/kg of *Nigella sativa*, the second group (CFA + *Nigella sativa* 1.82 mL/kg) was treated with 1.82 mL/kg of *Nigella sativa*, the third group (CFA) received 1.82 mL/kg of vehicle (saline) and the fourth group (CFA + indomethacin) was treated with 3 mg/kg of indomethacin as the reference drug. The indomethacin solution was prepared by dissolving the powder in saline and was given at a volume of 1.82 mL/kg. Oral treatments were given 1 h before the induction of arthritis on day-0 or 1 h before testing on the other days.

### 2.7. Body Weight Assessment

Body weight of rats was measured on days 0, 5, 15 and 25 and expressed as % change in body weight with respect to baseline weight (day 0).

### 2.8. Hind Paw Volume Assessment

The volumes of the injected and contralateral hind paws (measured to the hair-line) were assessed on days 0 (prior to the immunization with CFA), 5, 10, 15, 20 and 25 by a plethysmograph (Ugo Basile, Gemonio (VA), Italy). The increase in paw volume (ΔV) or edema at time (t) was calculated using the formula: Paw volume (t0) – paw volume (t), where the paw volume (t0) was measured on day 0 prior to immunization. Before volume measurement, animals were anesthetized with 3% isoflurane in oxygen.

### 2.9. Percentage Inhibition of Paw Edema

**Figure 1:**
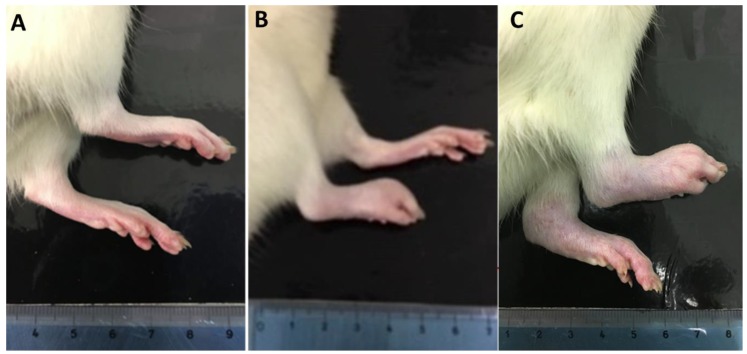
Changes in footpads after complete Freund’s adjuvant (CFA) immunization into the right hind paw of rat. (**A**) Control rat with no inflammation; (**B**) CFA-immunized rat with clinical signs of acute inflammation in the right hind paw and (**C**) CFA-immunized rat with arthritis that typically appears in the left non-injected hind paw about 15 days after CFA immunization.

Acute inflammation with appearance of primary arthritic lesions was observed in the injected hind paw (right) within 30 min of injection and often persisted for 20 to 25 days, whereas secondary arthritic lesions typically appeared in the non-injected paw (left) 15 days after sensitization and often persisted through 25 days (Figure 1).

To define if *Nigella sativa* treatment could prevent/reduce the appearance of secondary arthritic lesions when compared with arthritic rats (CFA group), the data of contralateral paw volume were analyzed by determining the area under the dosing curve (AUC) from day 0 to day 25. Means for each group were determined and % inhibition of paw edema with respect to the CFA group was calculated using the following formula: % inhibition of paw edema = B/A × 100, where A = mean of the healthy control group—mean of the CFA group and B = mean of the treated CFA group—mean of the CFA group. This method takes into account non-zero values for the healthy control group. Percentage inhibition close to 100% indicates effectiveness of treatment to prevent/reduce the incidence of arthritis compared to arthritic CFA group.

### 2.10. Arthritis Score

The severity of arthritis was scored by visual inspection in order to evaluate the ability to walk on days 5, 15 and 25. The scoring of gait behavior was performed by a modification of the previously reported scale [14]. Each animal was scored on a scale of 0 to 4 where: 0 = rat walks and runs normally; 1 = rat walks and runs with difficulty; 2 = rat shows limping without retraction of hind paw; 3 = rat shows limping with retraction of hind paw (does not touch the hind paw on the floor) and 4 = rat crawls or lies down only. The maximum score of every arthritic rat was 4. 

### 2.11. Spontaneous Locomotor Activity

On day 15, all animals were subjected to the open field test for 5 min in order to determine both spontaneous locomotor activity and anxiety-like behavior using automated locomotor activity boxes (Med Associates, San Diego, VT05478, USA). Ambulatory counts measured in the central and peripheral zones of open field provide information about the relative anxiety status of rats [15]. The procedures and parameters (ambulatory and vertical counts) measured in both tests were previously reported by Nasuti et al. [16].

### 2.12. Mechanical Allodynia

A dynamic plantar aesthesiometer (Ugo Basile, Germonio (VA), Italy) was used to determine mechanical allodynia by quantifying the withdrawal threshold of the hind paw in response to a mechanical stimulus, 1 h after administration of drugs on days 0, 5, 15 and 25. Rats were placed in elevated, clear plastic boxes (15 cm  ×  16  cm  ×  21  cm) with a wire-rung floor and allowed to acclimatize for 20 min immediately prior to the session. Mechanical stimulation (force of 0 to 50 g over a period of 20 s) through a metal filament was applied to the plantar surface of the hind paw. When the animal withdrew its hind paw, the automated device recorded the latency time. Animals were subjected to three consecutive trials for each hind paw with at least 5 min between trials and the average was calculated. A significant decrease in the latency time to elicit paw withdrawal in response to a mechanical stimulus was interpreted as mechanical allodynia.

### 2.13. Plasma IL-6, CRP, Albumin and TC Analysis

At day 15, blood from the tail vein of animals previously anesthetized with 3% isoflurane in oxygen was collected into tubes containing Ethylenediaminetetraacetic acid (EDTA) as an anticoagulant and immediately centrifuged (2000× g, 5 min). Plasma was collected and stored at −80 °C for analysis of IL-6, CRP, albumin and TC levels.

At the end of the experiment (day 25), all animals were euthanized by carbon dioxide inhalation and their blood, liver and brain tissues were collected. Blood samples were collected by cardiac puncture into tubes containing EDTA and centrifuged (2000 × g, 5 min) at 4 °C. Plasma was collected and stored at −80 °C until analysis. Liver and brain were immediately frozen in liquid nitrogen and stored at −80 °C.

IL-6 and CRP levels in the plasma of rats were quantified by the ELISA method using commercially available kits (Invitrogen, Milano, Italy), according to the manufacturer’s instructions. Analyses were performed on a Fluostar Omega microplate reader (BMG Labtech, Ortenberg, Germany). Plasma albumin levels in the samples were determined spectrophotometrically at 628 nm with the bromocresol green method [17] using a commercially available kit (Chema Diagnostica, Monsano (AN), Italy). TC levels in the plasma were determined spectrophotometrically at 510 nm with the cholesterol oxidase enzymatic method [18] using a commercially available kit (Chema Diagnostica, Monsano (AN), Italy). All assays were performed in duplicates. The statistical comparison among groups was performed by one-way ANOVA followed by Newman–Keuls post-hoc test.

### 2.14. Clustering Analysis of Haematological Parameters

K-medoids clustering analysis was applied to all hematological data collected on day 15 for comparing the group with a higher inflammatory response with the one showing a lower inflammatory response such as the healthy control group. The K-medoids clustering algorithm is an unsupervised learning technique for dividing a data set in k clusters. Each cluster in the K-medoids clustering approach is represented by one of the data points within the cluster and it is called a medoid. A medoid is an object within a cluster with the property that the average dissimilarity between it and all the other points of the same cluster is minimal. In practical terms, it represents the most centrally located point within the cluster. Each cluster is represented by its medoid. One of the strengths of the K-medoids clustering algorithm compared to the more classic K-means, is its robustness: The fact that in the latter the centroid of the cluster is calculated as the midpoint between all the points in the cluster makes it particularly sensitive to noise and outliers.

The quality of clusters was evaluated through the average silhouette: This tells us how well each object fitted within its cluster. In the average silhouette method, we computed the average silhouette of the data for different values of k; then, the optimal number of clusters, k, was the one that was able to maximize the average shape with respect to an interval of k. In this work we used the free software R for the analysis, where the K-medoids clustering algorithm was implemented in the partitioning around medoids algorithm [19].

### 2.15. Statistical Analysis

Data are expressed as means ± SD and evaluated using one-way ANOVA (Statistica 8.0, StatSoft, Tulsa, OK, USA). Two-way repeated measures ANOVA test was used where applicable. Newman–Keuls post hoc test was used when appropriate. Differences were considered significant at a *p*-value < 0.05.

## 3. Results

### 3.1. Fatty Acids and Tocopherols Levels in Nigella sativa Oil

*Nigella sativa* oil was analyzed for its fatty acid content; the composition of the oil produced in the Marche Region (Italy) resulted to be similar to those produced in other Mediterranean countries (i.e., Morocco, Egypt and Tunisia) [20]. Specifically, about 80% of the unsaturated fatty acids were linoleic and oleic acids, while palmitic acid was the main saturated one as shown in Table 1.

Tocopherols were mainly constituted by the γ-form (103.9 mg/kg), followed by the α-form (26.6 mg/kg) as reported from previous studies on cold pressed oil of *Nigella sativa*. However, the content of γ-tocopherol we measured was 103.9 mg/kg against the lower level ranged between 9.7–45.1 mg/kg reported by Matthaus et al. [21].

### 3.2. Safety Profile of Nigella sativa Oil in Rats

In the acute toxicity study, *Nigella sativa* oil did not lead to any death nor to any visible symptoms of toxicity in rats within the first 24 h and for the further 14 days at the highest dose of 2000 mg/kg body weight employed. The oral LD50 for *Nigella sativa* oil was more than 2000 mg/kg and the oil could be included in category 5 of the globally harmonized system. Toxicity signs such as piloerection, salivation, lacrimation, convulsion, tremor, diarrhea, lethargy and changes in body weight were not observed during the 14 days of observation. Histopathological examination of organs did not show any pathological changes (data not shown). Since the extract was safe up to 2000 mg/kg body weight, one fifth of this dose (i.e., 1596 mg/kg body weight corresponding to 1.82 mL/kg body weight) was chosen as the highest dose for pharmacological studies. The present research study was carried out using two different doses of *Nigella sativa* oil: 1.82 and 0.91 mL/kg body weight for the evaluation of anti-arthritic activity in an AIA model.

### 3.3. Body Weight Changes in CFA-Induced Arthritic Rats 

**Figure 2:**
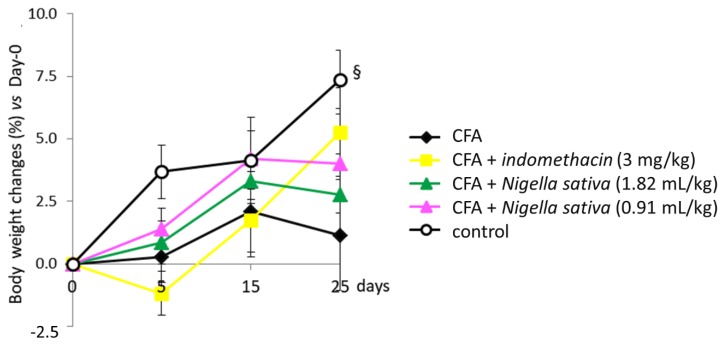
Effect of *Nigella sativa* oil on disease progression in CFA-induced arthritic rats. CFA-immunized rats (on day 0) received *Nigella sativa* oil (1.82 or 0.91 mL/kg), indomethacin (3 mg/kg) or vehicle by gastric gavage daily for 25 days. A group of non-immunized rats receiving vehicle during the same period served as a healthy control. Body weight was measured prior to the immunization, on day 0, and up to 25 days. Data expressed as mean ± SD were analyzed using two-way repeated measures ANOVA; *n* = 12 rats per group except control group with *n* = 11 rats. § *p* < 0.05 vs. all groups.

The body weight at the first evaluation (day 0 prior the immunization) was almost identical among all groups of animals, while it was always significantly lower in the immunized rats than in the control ones during the subsequent period (Figure 2). During the last week, the weight of immunized rats tended to return towards that of control rats.

### 3.4. Anti-Inflammatory Activity of Nigella sativa Oil in CFA-Induced Arthritic Rats

**Figure 3:**
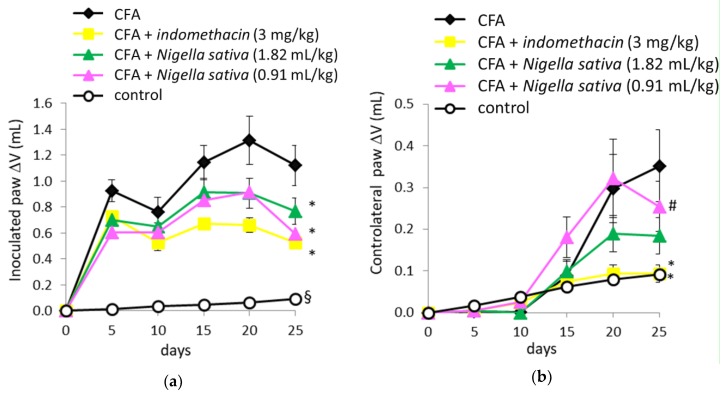
Effect of *Nigella sativa* oil on disease progression in CFA-induced arthritic rats. CFA-immunized rats (on day 0) received *Nigella sativa* oil (1.82 or 0.91 mL/kg), indomethacin (3 mg/kg) or vehicle by gastric gavage daily for 25 days. A group of non-immunized rats receiving vehicle during the same period served as a healthy control. Measurement of edema in the inoculated (**a**) and controlateral (**b**) paws was performed prior to the immunization, on day 0, and up to 25 days. Data expressed as mean ± SD were analyzed using two-way repeated measures ANOVA; *n* = 12 rats per group except control group with *n* = 11 rats. * *p* < 0.05 vs. CFA group; # *p* < 0.05 vs. control group; § *p* < 0.05 vs. all groups.

When volume changes of an inoculated paw (right) were analyzed by ANOVA for repeated measures, the results showed significant effects in the treatment groups (F[4, 54] = 22.4, *p* < 0.001), time (F[4, 216] = 15.2, *p* < 0.001) and a significant treatment by time interaction (F[16, 216] = 2.9, *p* < 0.001), as reported in Figure 3a. Post hoc Newman–Keuls test demonstrated a significant increase in paw volume in all groups inoculated with CFA when compared with the control group (*p* < 0.05). Both groups treated with *Nigella sativa* (1.82 and 0.91 mL/kg) together with the CFA + indomethacin group showed a significant reduction in paw volume (*p* < 0.05) when compared with the CFA group. When volume changes of the contralateral paw (left) were analyzed, ANOVA for repeated measures revealed significant effects in the treatment groups (F[4, 54] = 3.3, *p* < 0.05), time (F[4, 216] = 39.2, *p* < 0.001), and a significant treatment × time interaction (F[16, 216] =3.6, *p* < 0.001) as reported in Figure 3b. Post hoc Newman–Keuls test demonstrated a significant increase in paw volume in both CFA and CFA + *Nigella sativa* (0.91 mL/kg) groups when compared with the control group (*p* < 0.05)

**Figure 4:**
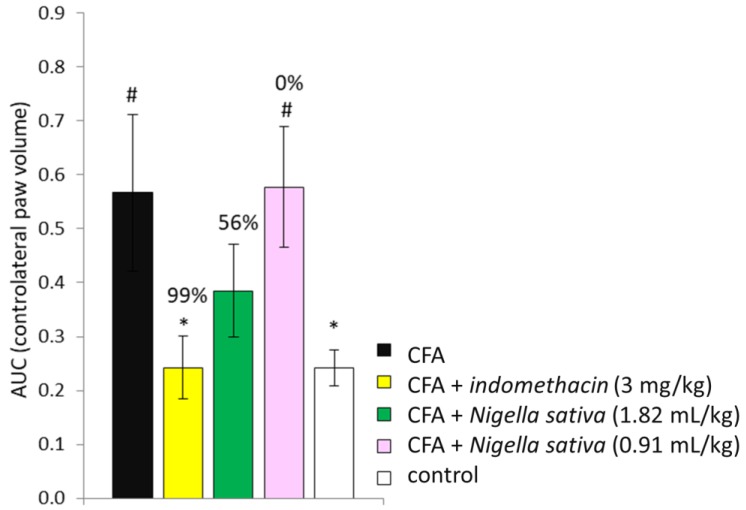
Effect of *Nigella sativa* oil on % inhibition of arthritis in CFA-induced arthritic rats. CFA-immunized rats (day 0) received *Nigella sativa* oil (1.82 or 0.91 mL/kg), indomethacin (3 mg/kg) or vehicle by gastric gavage daily for 25 days. A group of non-immunized rats receiving vehicle during the same period served as a healthy control. Data of contralateral paw volume over time expressed as area under the curve (AUC) was used to calculate % inhibition from the arthritic control. Data expressed as mean ± SD were analyzed using a one-way ANOVA for panel A and two-way repeated measures ANOVA for panel B; *n* = 12 rats per group except control group with *n* = 11 rats. * *p* < 0.05 vs. CFA group; # *p* < 0.05 vs. control group.

The treatment with indomethacin was effective to reduce the paw volume *(p* < 0.05) when compared with the CFA group, whereas only a trend towards anti-inflammatory activity at the high dose of *Nigella sativa* (1.82 mL/kg) was observed *(p* > 0.05). No anti-inflammatory activity was observed in CFA-induced rats treated with a low dose of *Nigella sativa* (0.91 mL/kg). Expression of the data (contralateral paw volume over time) as AUC (area under the curve) and the calculation of percentage inhibition from arthritic control rats (CFA group) showed that indomethacin was effective to reduce paw swelling throughout the study *(p <* 0.05 vs. CFA group), whereas *Nigella sativa* at a high dose (1.82 mL/kg) was effective only by trend *(p* > 0.05 vs. CFA group) as reported in Figure 4. The % inhibition of arthritis in the CFA + indomethacin group was 99% and 56% in CFA + *Nigella sativa* (1.82 mL/kg), whereas the low dose of *Nigella sativa* failed to produce any positive effect in CFA-injected rats.

### 3.5. Effect of Nigella sativa Oil on the Arthritis Score in CFA-Induced Arthritic Rats

**Figure 5:**
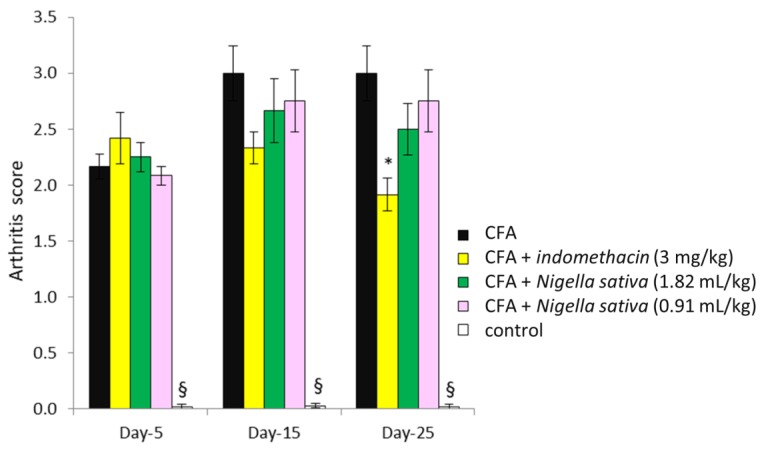
Effect of *Nigella sativa* oil on the arthritis score in CFA-induced arthritic rats. CFA-immunized rats (day 0) received *Nigella sativa* oil (1.82 or 0.91 mL/kg), indomethacin (3 mg/kg) or vehicle by gastric gavage daily for 25 days. A group of non-immunized rats receiving vehicle during the same period served as a healthy control. Arthritis score was measured on days 5, 15 and 25. Data expressed as mean ± SD were analyzed using a one-way ANOVA for panel A and two-way repeated measures ANOVA for panel B; *n* = 12 rats per group except the control group with *n* = 11 rats. * *p* < 0.05 vs. CFA group; § *p* < 0.05 vs. all groups.

The scoring of mobility was analyzed by two-way repeated measures ANOVA; the results revealed significant effects of treatment (F[4, 54] = 53.05, *p* < 0.001), time (F[2, 108] = 7.9, *p* < 0.001) and treatment × time interaction (F[8, 108] = 3.6, *p* < 0.001), as reported in Figure 5. Post hoc Newman–Keuls test demonstrated a significant increase in arthritis score in all groups inoculated with CFA when compared with the control group (*p* < 0.05). No significant differences were detected among groups inoculated with CFA (*p* > 0.05), except for day 25, where the arthritis score in the CFA + indomethacin group was lower than that detected in the CFA group (*p* < 0.05).

### 3.6. Effect of Nigella sativa Oil on Spontaneous Locomotor Activity in CFA-Induced Arthritic Rats

In the open field test, ANOVA revealed no differences in horizontal (F[4, 54] = 0.48, *p* > 0.05) and vertical (F[4, 54] = 0.33, *p* > 0.05) activities among groups. The ambulatory counts were 1539.91 ± 166.19, 1730.16 ± 127.21, 1678.45 ± 127.95, 1634.42 ± 222.84 and 1633.83 ± 74.53 for CFA, CFA + indomethacin, CFA + *Nigella sativa* 1.82 mL/kg, CFA + *Nigella sativa* 0.91 mL/kg and control groups, respectively. The vertical counts were 51.27 ± 4.99, 60.66 ± 4.18, 50.36 ± 6.79, 53.75 ± 4.95 and 58.33 ± 6.42 for CFA, CFA + indomethacin, CFA + *Nigella sativa* 1.82 mL/kg, CFA + *Nigella sativa* 0.91 mL/kg and control groups, respectively. For anxiety-like behavior, the ANOVA revealed an absence of differences in locomotor activity expressed as % ambulatory counts (F[4, 54] = 0.73, *p* > 0.05) and % vertical counts (F[4, 54] = 1.68, *p* > 0.05) occurring in the central area of the open field among groups (supplementary Table S1). Normal locomotor activity and a lack of anxiety in CFA-treated rats indicate that the animal model of RA used here, shows reduced distress and low severity of disease.

### 3.7. Anti-Nociceptive Activity of Nigella sativa Oil in CFA-Induced Arthritic Rats

**Figure 6:**
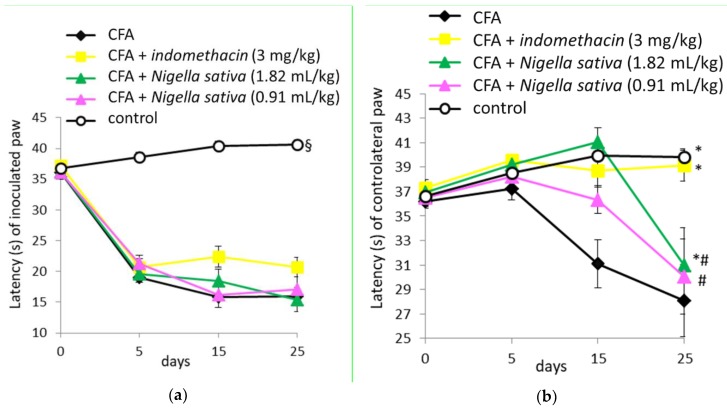
Effect of *Nigella sativa* oil on disease progression in CFA-induced arthritic rats. CFA-immunized rats (on day 0) received *Nigella sativa* oil (1.82 or 0.91 mL/kg), indomethacin (3 mg/kg) or vehicle by gastric gavage daily for 25 days. A group of non-immunized rats receiving vehicle during the same period served as a healthy control. Evaluation of mechanical allodynia in the inoculated (**a**) and controlateral (**b**) paws was performed prior to the immunization, on day 0, and up to 25 days. Data expressed as mean ± SD were analyzed using two-way repeated measures ANOVA; *n* = 12 rats per group except the control group with *n* = 11 rats. * *p* < 0.05 vs. CFA group; # *p* < 0.05 vs. control group; § *p* < 0.05 vs. all groups.

On day 0, the paw withdrawal latency in CFA-injected rats was not significantly different from that in the control group in either inoculated or contralateral paws (*p* > 0.05) as reported in Figure 6a,b, respectively.

When the withdrawal latency of the inoculated paw (right) was analyzed by two-way repeated measures ANOVA, the results revealed significant effects for treatment groups (F[4, 54] = 55.3, *p* < 0.001), time (F[3, 162] = 176.7, *p* < 0.001) and a significant treatment by time interaction (F[12, 162] = 17.5, *p* < 0.001), as reported in Figure 6a. Post hoc Newman–Keuls test demonstrated a significant decrease in paw withdrawal latency in all groups inoculated with CFA when compared with the control group (*p* < 0.05). No difference was detected among groups inoculated with CFA (*p* > 0.05). Both indomethacin and *Nigella sativa* treatments did not have anti-nociceptive activity in the inoculated hind paw, which is a primary arthritic lesion with higher sensitivity to pain compared to the controlateral one.

When withdrawal latency of the controlateral paw (left) was analyzed by two-way repeated measures ANOVA, the results revealed significant effects for treatment (F[4, 54] = 5.9, *p* < 0.001), time (F[3, 162] = 11.3, *p* < 0.001) and treatment × time interaction (F[12, 162] = 3.9, *p* < 0.001), as reported in Figure 6b. Post hoc Newman–Keuls test demonstrated a significant decrease in paw withdrawal latency in the CFA group when compared with the control, CFA + indomethacin and CFA + *Nigella sativa* (1.82 mL/kg) groups (*p* < 0.05). Conversely, the latency in the CFA + *Nigella sativa* (0.91 mL/kg) group was similar to that in the CFA group (*p* > 0.05) and lower than those detected in the control and CFA + indomethacin groups (*p* < 0.05). Both indomethacin and *Nigella sativa* (1.82 mL/kg) treatments had anti-nociceptive activity in the controlateral hind paw, which is a secondary arthritic lesion with lower sensitivity to pain compared to the inoculated one.

### 3.8. Effect of Nigella sativa Oil on Plasma IL-6, CRP, Albumin and TC Levels in CFA-Induced Arthritic Rats

For plasma IL-6 levels, differences among groups were observed on day 15 (F[4, 54] = 13.66, *p* < 0.001) as shown in Table 2. 

In particular, IL-6 levels were significantly higher (*p* < 0.05) in arthritic (CFA group) versus control rats on day 15, whereas they had returned to normal values (such as control healthy rats) on day 25 (*p* > 0.05). The treatment with indomethacin was effective to reduce IL-6 levels in plasma of CFA-induced rats (*p* < 0.05 versus CFA group), whereas the treatment with *Nigella sativa* oil (both doses) failed to produce any significant effect (*p* > 0.05 versus CFA group). No significant differences in IL-6 levels were observed among groups on day 25 (F[4, 54] = 0.72, *p* > 0.05).

With regard to plasma CRP levels, a one-way ANOVA revealed significant effects of treatment on day 15 (F[4, 54] = 11.58, *p* < 0.001) as reported in Table 2. CRP levels in both CFA + *Nigella sativa* (1.82 mL/kg) and CFA + *Nigella sativa* (0.91 mL/kg) groups were significantly higher than those measured in all groups on day 15, whereas they had returned to normal values (such as control healthy rats) on day 25 (*p* > 0.05). No significant differences in CRP levels were observed among CFA, CFA + indomethacin and control groups (*p* > 0.05) on days 15 and 25. 

Plasma albumin levels were significantly decreased in all groups inoculated with CFA when compared with healthy control rats at both 15 and 25 days (F[4, 54] = 9.69, *p* < 0.001 and F[4, 54] = 4.43, *p* < 0.01, respectively) as shown in Table 2.

No significant differences among groups were observed in plasma TC levels on day 15 (F[4, 54] = 0.89, *p* > 0.05), whereas both CFA + *Nigella sativa* (1.82 mL/kg) and CFA + *Nigella sativa* (0.91 mL/kg) groups had significantly lower TC levels compared to all groups on day 25 (F[4, 54] = 3.78, *p* < 0.01).

**Figure 7:**
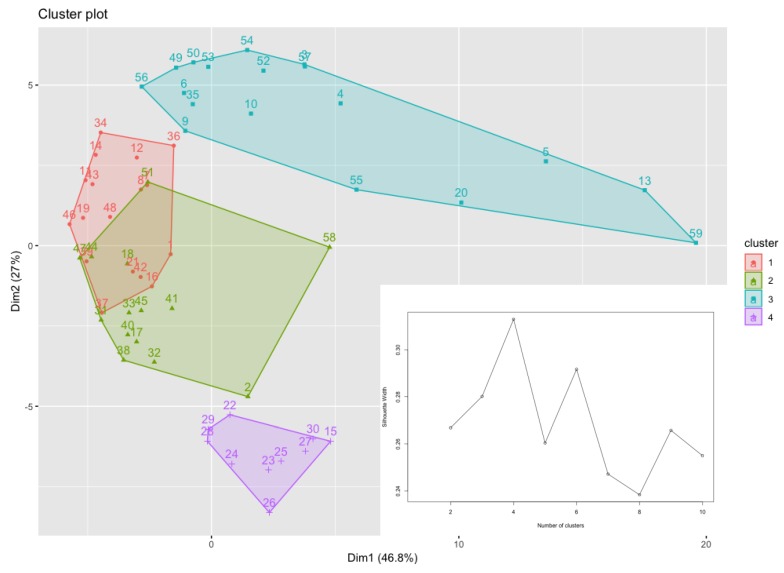
Clustering analysis applied to hematological data collected from all rats on day 15. Data points representing all rat samples are separated into four differently colored clusters using k-Medoids clustering algorithm. Cluster 3 is composed of the control, CFA + indomethacin and some rats of CFA groups. Cluster 4 only includes rats belonging to CFA + *Nigella sativa* and CFA groups. Both groups treated with *Nigella sativa* oil are distributed in clusters 1, 2 and 3. The inset of the figure represents the average silhouette function indicating the optimal number of clusters.

Cluster analysis applied to all hematological parameters led to the clustering of 59 rats into four major groups called clusters 1, 2, 3 and 4 consisting of 17, 14, 18 and 10 rats respectively, as reported in Figure 7.

The same figure also shows the average silhouette function where it is possible to notice that the optimal number of clusters was four. Cluster 3 was composed of healthy rats with lower inflammatory response such as the control and CFA + indomethacin groups and two rats of CFA group. Cluster 4 corresponded to a more diffuse pattern of arthritis and included the arthritic CFA group and some rats treated with *Nigella sativa* oil (1.82 kg/mL). Clusters 1 and 2 contained rats of all treatments.

## 4. Discussion

*Nigella sativa* is a plant broadly used in traditional medicine for inflammatory conditions such as RA, but there are few scientific studies supporting the claimed use [22,23]. In this study, we attempted to evaluate the effect of the crude oil extracted from *Nigella sativa* seeds produced in the Marche region (Central Italy) in adjuvant-induced arthritic rats.

The results on the acute toxicity study suggested that *Nigella sativa* oil has a large window of safety with a LD50 > 2000 mg/kg, as previously reported [24,25]. On this basis, two doses of *Nigella sativa* oil were chosen for testing: 1.82 and 0.91 mL/kg corresponding to 1596 and 798 mg/kg, respectively. The animals were treated orally with the plant extract or indomethacin for 25 days starting from the day of immunization (day 0). Changes in body weight of rats were measured as one of the parameters to assess the course of disease since weight loss indicates active disease, whereas weight gain is indicative of recovery. The lower weight gain observed in all groups immunized with respect to the control group was in accordance with the fact that RA was associated with the loss of lean body mass, known as rheumatoid cachexia, a key comorbidity. The latter is thought to be the final outcome of cytokine-driven hypermetabolism, which, in turn, increases the activity of the ubiquitin-proteasome proteolytic pathway responsible for muscle wasting and hence weight loss [26]. Despite the lower body weight gain observed in all animals inoculated with CFA, in the last period of the experiment (3rd week), the treatments with *Nigella sativa* oil or indomethacin seemed to minimize the loss in body mass. In fact, CFA-treated animals receiving *Nigella sativa* oil or indomethacin showed a more similar trend to the healthy control group compared with CFA-untreated group.

CFA-induced arthritis is a chronic inflammation characterized by two phases: An acute phase (from day 0) with local inflammatory reactions that give rise to primary arthritic lesions such as edema in the injected paw, followed by a chronic inflammatory phase (from day 15) with secondary arthritic lesions characterized by inflammatory edema in the controlateral paw [27,28]. The first acute phase is caused by various mediators such as cytokines IL-1β and TNF-α, prostaglandins, histamine, kinins and serotonin released by leukocytes that migrate to the affected region and are responsible for edema. The secondary chronic phase is a manifestation of cell-mediated autoimmunity and cytokine IL-6 is the principal mediator involved in the cartilage and bone degenerative process.

Arthritis progression and anti-arthritic activity of drugs can be assessed through the measurement of several parameters such as volumes of both hind paws and arthritis score. Swelling of the injected hind paw (primary arthritic lesion) was used for evaluating the inflammatory response in the acute phase following CFA immunization while the increased volume of the controlateral hind paw (secondary arthritic lesion) was measured to estimate the generalized immune response leading to polyarthritis in the chronic phase. The animal groups that were given either indomethacin or *Nigella sativa* oil showed a marked reduction in the injected hind paw volume when compared with the arthritic CFA group. This result suggests that both doses of *Nigella sativa* oil were effective in reducing inflammation in the acute phase of RA. However, the same positive effect of the *Nigella sativa* treatment was not observed in the controlateral paw. Conversely, indomethacin was also effective in the secondary phase of inflammation, markedly reducing the volume of the contralateral paw and demonstrating a strong 99% inhibitory action against inflammation. 

Despite that treatment with *Nigella sativa* was not effective in the secondary phase of inflammation, suppression of the inflammatory response in the initial phase by the higher dose prevented the development of arthritis with an inhibition of 56%. On the other hand, the low-dose prophylactic treatment did not prevent the onset of arthritis. Arthritis is associated with functional impairment of walking ability that was measured by the arthritis score. Rats injected with CFA and developing RA showed abnormal gait behavior (i.e., limping up to crawling in the animals with high disability) with increased arthritis score. The treatment with the plant extract at both doses did not improve the ability to walk, whereas the positive effect in rats treated with indomethacin was observed at the end (on day 25) showing significant improvement in the arthritis score compared to CFA group. 

These results indicate that *Nigella sativa* oil reduced local inflammation in the acute phase and prevented the development of RA but was not able to ameliorate the severity of arthritis in the chronic phase. Although we did not come across any study evaluating the anti-inflammatory activity of *Nigella sativa* oil in an AIA animal model, Al-Ghamdi demonstrated the anti-inflammatory effect of an aqueous *Nigella sativa* extract (500 mg/kg per o.s.) in a carrageenan animal model of acute inflammation [29]. Similar observations on formalin-induced inflammation in rats treated with an ethanol extract of *Nigella sativa* have been reported by Tanko et al. [30].

Of interest is the fact that when arthritic rats were submitted to an open field test, they displayed a similar spontaneous locomotor activity as the healthy control rats. Although the arthritic rats had abnormal gait behavior, such as limping, they did not decrease their locomotor activity, suggesting the development of mild arthritis in our animal model. This was further confirmed by the absence of anxiety-like behavior and minimal loss in body weight (no more than 3% versus control rats) in arthritic rats. We might assume that the low volume of CFA (10 μL) injected into the footpad minimized the suffering of animals and gave rise to less severe arthritis. One of the goals of the present study was to evaluate the potential anti-nociceptive effect of *Nigella sativa* oil on arthritic rats submitted to mechanical allodynia. The plant extract revealed an earlier significant anti-nociceptive activity in arthritic rats only at the highest dose, with a peak activity on day 15, suggesting a good anti-inflammatory and anti-arthritic activity during the acute phase; thereafter, a decrease in anti-nociceptive activity was observed. Conversely, the anti-nociceptive activity of indomethacin persisted during the chronic phase such as the anti-inflammatory activity as discussed above. This result was in agreement with the lower level of circulating IL-6 measured as early as day 15 in the CFA + indomethacin group compared to CFA group. At day 15, indomethacin reduced the increase in IL-6 caused by RA induction to levels observed in control healthy rats, whereas *Nigella sativa* oil failed to reduce IL-6 levels and did not show anti-nociceptive and anti-inflammatory activity during the chronic phase of RA. The plasmatic profile of IL-6 in CFA and CFA + indomethacin groups reported here, is similar to those published by other authors where IL-6 appears as the plasmatic indicator of the chronic phase and its pattern parallels the time-course of the clinical changes observed on paw edema and arthritic pain [31]. In addition, inhibition of systemic production of IL-6 by treatment with indomethacin is well documented and accounts for the fact that cyclooxygenase-derived prostaglandin mediated IL-6 production [32,33,34]. Non-specific inflammatory markers such as CRP, albumin and TC were measured in plasma as indices of inflammatory status over the course of RA; CRP [35,36] and TC [37] plasma levels seem to be positively correlated with inflammation, whereas albumin plasma levels are negatively correlated [38].

In line with the increased IL-6 levels measured in CFA rats treated with *Nigella sativa* oil (1.82 or 0.91 mL/kg) at day-15, both groups showed increased CRP levels with respect to control healthy rats. These findings rule out the possibility that *Nigella sativa* treatment may have the capacity to block the inflammatory response during RA progression through the inhibition of inflammatory mediators such as IL-6 and CRP. Although there is no study evaluating *Nigella sativa* oil in animal models of RA, several authors demonstrated its efficacy in rheumatic patients through increased anti-inflammatory cytokine IL-10 levels and decreased oxidative stress parameters in plasma without affecting IL-6 levels [39,40]. On the other hand, a study on a CIA animal model showed that thymoquinone, the main component of *Nigella sativa* oil, neutralized free radicals and then reduced the plasmatic IL-6 levels after 21 days of treatment [22]. Moreover, our previous in vitro study reported antioxidant and anti-inflammatory properties of *Nigella sativa* oil in low-grade inflammation human pre-adipocytes [10]. In particular, the same extract here studied, was able to decrease IL-6 levels after 24 h of incubation with pre-adipocytes.

With regards to albumin levels in plasma, all groups CFA-injected showed lower values compared to healthy control group suggesting that the inflammatory process was still not over on day 25 as it was seen in clinical evaluations.

In the light of previous evidences suggesting a hypocholesterolemic effect of *Nigella sativa* [41] and considering the positive effect of hypolipidemic drugs in RA [42], we decided to measure TC plasma levels in rats. The CFA group did not show any increase in TC levels with respect to control healthy rats. Contradictory observations concerning lipid profiles in plasma of RA patients have been reported: Lakatos et al. [43] described higher levels of TC, Lazarevic et al. [44] found lower TC levels and Dorsunoglu et al. [45] noticed no changes in TC levels. Of interest is the fact that the treatment with both doses of *Nigella sativa* oil lowered plasma TC levels in arthritic rats, at day 25, when inflammatory markers such as CRP and IL-6 levels returned to those of control healthy rats. Conversely, at day 15, the same groups showed higher CRP and IL-6 levels compared to control rats and TC levels were similar to those of control rats. The results here obtained, suggest that *Nigella sativa* treatment lowers the levels of TC, which, in turn, decrease the degree of inflammation, restoring normal CRP and IL-6 levels. A similar effect was described in arthritic patients treated with statins, where a lower degree of inflammation with clinical improvements in RA was reported in conjunction with decreased plasma TC and CRP levels [42].

K-medoids clustering analysis applied to all hematological data collected on day 15 identified 4 categories: Cluster 3 was composed mostly of control and CFA + indomethacin samples, clusters 1, 2 and 4 included those related to CFA group, and in all these clusters, samples belonging to both CFA + *Nigella sativa* groups were found. It is worth mentioning that the IL-6 values were very consistent in the cluster despite the treatment. It decreased from cluster 4 to cluster 3 (following the positive direction of the Principal Component (PC). In agreement with the previous clinical results, indomethacin was the most effective treatment, followed by *Nigella sativa* at highest dose, while the lowest dose of the oil was the least effective.

## 5. Conclusions

In conclusion, the present findings supported the hypothesis that the anti-inflammatory, anti-arthritic and anti-nociceptive effect of *Nigella sativa* oil administered sub-chronically on AIA rats was dose-dependent, and that the higher dose (1.82 mL/kg) prevented the development of arthritis with an inhibition of 56%. In particular, *Nigella sativa* oil was effective in the control of acute phase inflammation and prevented the development of RA, but it was not able to ameliorate the severity of arthritis in the chronic phase where indomethacin resulted in being more active.

Despite the positive effects on clinical signs of RA, *Nigella sativa* oil did not improve plasma IL-6 levels, whereas it reduced plasma TC levels. By contrast, indomethacin improved IL-6 levels and did not change plasma TC levels. Overall, the data strongly suggested a potential control of cholesterol metabolism, which remains to be elucidated to better understand the beneficial effects of *Nigella sativa* oil in RA.

## Figures and Tables

**Table 1:** Fatty acid (%) and tocopherol composition (mg/kg) in *Nigella sativa* oil.

Fatty Acid and Tocopherol	Mean ± SD
Myristic acid (C14:0)	0.16 ± 0.01
Palmitic acid (C16:0)	12.80 ± 0.21
Palmitoleic acid (C16:1)	0.18 ± 0.01
Margaric acid (C17:0)	0.08 ± 0.001
Margaroleic acid (C17:1)	0.06 ± 0.001
Stearic acid (C18:0)	3.39 ± 0.01
Oleic acid (C18:1)	24.65 ± 0.02
Linoleic acid (18:2)	57.92 ± 0.19
Linolenic acid (C18:3)	0.22 ± 0.002
Arachidic acid (C20:0)	0.24 ± 0.01
Eicosenoic acid (C20:1)	0.30 ± 0.02
Saturated fatty acid (SFA)	16.67 ± 0.21
Monounsaturated fatty acid (MUFA)	25.19 ± 0.03
Polyunsaturated fatty acid (PUFA)	58.14 ± 0.19
α-Tocopherol	26.60 ± 1.60
γ-Tocopherol	103.90 ± 5.90

Data are expressed as mean ± standard deviation of two replicates.

**Table 2:** Effect of *Nigella sativa* oil on interleukin-6 (IL-6), C-reactive protein (CRP), albumin and total cholesterol (TC) levels in plasma of CFA-induced arthritic rats.

		CFA *n* = 12 rats	CFA + Indomethacin (3 mg/kg) *n* = 12 rats	CFA + *Nigella sativa* (1.82 mL/kg) *n* = 12 rats	CFA + *Nigella* *sativa* (0.91 mL/kg) *n* = 12 rats	Control *n* = 11 rats
**IL-6**	Day 15	465.12 ± 136.87	310.24 ± 100.10 *	522.86 ± 148.47 ^#^	418.21 ± 25.98 ^#^	249.70 ± 26.02 *
**(pg/mL)**	Day 25	241.67 ± 90.14	218.33 ± 77.76	254.05 ± 54.60	258.46 ± 44.22	243.17 ± 26.14
**CRP**	Day 15	3.23 ± 1.63	2.45 ± 0.54	4.91 ± 0.90 ^##^	4.49 ± 0.43 ^##^	2.90 ± 1.36
**(mg/mL)**	Day 25	3.29 ± 0.55	2.30 ± 1.02	2.80 ± 0.80	2.71 ± 0.58	2.91 ± 1.36
**Albumin**	Day 15	2.06 ± 0.35	2.05 ± 0.44	2.24 ± 0.30	2.24 ± 0.28	2.81 ± 0.30 ^§^
**(g/dL)**	Day 25	2.41 ± 0.29	2.19 ± 0.74	2.19 ± 0.25	2.21 ± 0.37	2.82 ± 0.30 ^§^
**TC**	Day 15	58.05 ± 11.18	58.06 ± 12.05	59.69 ± 7.89	64.72 ± 8.05	60.00 ± 10.73
**(mg/dL)**	Day 25	66.49 ± 12.13	62.73 ± 15.65	51.12 ± 10.55 *	52.36 ± 9.53 *	61.04 ± 10.74

Data expressed as mean ± standard deviation were analyzed using a one-way ANOVA. * *p* < 0.05 vs. CFA group; # *p* < 0.05 vs. control group; ## *p* < 0.01 vs. control group; § *p* < 0.05 vs. all groups.

## Supplementary Materials

The following are available online at https://www.mdpi.com/2076-3921/8/9/342/s1, Table S1: Effect of *Nigella sativa* oil on anxiety-like behavior of open filed test in CFA-induced arthritic rats.
