# Supplementary material for: Anti-Inflammatory, Anti-Arthritic and Anti-Nociceptive Activities of Nigella sativa Oil in a Rat Model of Arthritis
Source: Antioxidants (Basel). 2019 Aug 25;8(9):342. doi: 10.3390/antiox8090342 (PMC6769720; doi:10.3390/antiox8090342)
Supplement: Supplementary file 1 [file antioxidants-08-00342-s001.pdf]

Supplementary Table S1. Effect of *Nigella sativa* oil on anxiety-like behavior of open filed test in CFA-induced arthritic rats.

	CFA<br>n= 12 rats	CFA+Indo-methacin (3 mg/kg)<br>n= 12 rats	CFA+*Nigella sativa* (1.82 mL/kg)<br>n= 11 rats	CFA+*Nigella sativa* (0.91 mL/kg)<br>n= 12 rats	Control<br>n= 12 rats
% Amb. Cnts	3.97±2.01	3.67±1.80	4.46±2.10	4.70±2.01	4.73±1.67
% Vert. Cnts	1.65±2.23	1.28±1.87	1.76±2.32	1.98±1.43	1.63±1.32

% Amb. Cnts= percentage ambulatory counts; % Vert. Cnts = percentage vertical counts. Data expressed as mean ± standard deviation were analysed using one-way ANOVA. $P>0.05$.
